# Autophagy-lysosome pathway associated neuropathology and axonal degeneration in the brains of alpha-galactosidase A-deficient mice

**DOI:** 10.1186/2051-5960-2-20

**Published:** 2014-02-14

**Authors:** Michael P Nelson, Tonia E Tse, Darrel B O’Quinn, Stefanie M Percival, Edgar A Jaimes, David G Warnock, John J Shacka

**Affiliations:** Department Pathology, Neuropathology Division, University of Alabama at Birmingham, Birmingham, AL USA; Birmingham VA Medical Center, Birmingham, AL USA; Department Pathology, Anatomic Pathology Division, University of Alabama at Birmingham, Birmingham, AL USA; Department Pharmacology and Toxicology, University of Alabama at Birmingham, Birmingham, AL USA; Department Medicine, Nephrology Division, University of Alabama at Birmingham, Birmingham, AL USA

**Keywords:** α-Galactosidase A, α-synuclein, Brain, Neurodegeneration, Neuropathology, Immunohistochemistry, Electron microscopy

## Abstract

**Background:**

Mutations in the gene for alpha-galactosidase A result in Fabry disease, a rare, X-linked lysosomal storage disorder characterized by a loss of alpha-galactosidase A enzymatic activity. The resultant accumulation of glycosphingolipids throughout the body leads to widespread vasculopathy with particular detriment to the kidneys, heart and nervous system. Disruption in the autophagy-lysosome pathway has been documented previously in Fabry disease but its relative contribution to nervous system pathology in Fabry disease is unknown. Using an experimental mouse model of Fabry disease, alpha-galactosidase A deficiency, we examined brain pathology in 20-24 month old mice with particular emphasis on the autophagy-lysosome pathway.

**Results:**

Alpha-galactosidase A-deficient mouse brains exhibited enhanced punctate perinuclear immunoreactivity for the autophagy marker microtubule-associated protein light-chain 3 (LC3) in the parenchyma of several brain regions, as well as enhanced parenchymal and vascular immunoreactivity for lysosome-associated membrane protein-1 (LAMP-1). Ultrastructural analysis revealed endothelial cell inclusions with electron densities and a pronounced accumulation of electron-dense lipopigment. The pons of alpha-galactosidase A-deficient mice in particular exhibited a striking neuropathological phenotype, including the presence of large, swollen axonal spheroids indicating axonal degeneration, in addition to large interstitial aggregates positive for phosphorylated alpha-synuclein that co-localized with the axonal spheroids. Double-label immunofluorescence revealed co-localization of phosphorylated alpha-synuclein aggregates with ubiquitin and LC3.

**Conclusion:**

Together these findings indicate widespread neuropathology and focused axonal neurodegeneration in alpha-galactosidase A-deficient mouse brain in association with disruption of the autophagy-lysosome pathway, and provide the basis for future mechanistic assessment of the contribution of the autophagy-lysosome pathway to this histologic phenotype.

**Electronic supplementary material:**

The online version of this article (doi:10.1186/2051-5960-2-20) contains supplementary material, which is available to authorized users.

## Background

Alpha-Galactosidase A (α-Gal A) is a soluble lysosomal enzyme that hydrolyzes the terminal alpha-galactosyl moiety from glycolipids and glycoproteins. The predominant lipid hydrolyzed by α-Gal A is ceramide trihexoside, also known as globotriaosylceramide or Gb_3_[1]. Mutations in the α-Gal A gene (*GLA*) occur in the rare, X-linked lysosomal storage disorder called Fabry disease, and resultant decreases in α-Gal A enzymatic activity lead to the progressive and widespread accumulation of glycosphingolipids in most bodily tissues and fluids including Gb_3_ and globotriaosylsphingosine (also known as lyso-Gb_3_) [1, 2]. The prominent effects of α-Gal A deficiency and glycosphingolipid accumulation on the vascular endothelium in particular have long associated Fabry disease as a vasculopathy with resultant life-threatening complications to the kidneys, heart and brain (reviewed in [3]).

There are widespread central and peripheral nervous system manifestations of Fabry disease. Peripheral nervous system involvement includes small fiber neuropathy that is associated with neuropathic pain and autonomic dysfunction (reviewed in [4, 5]). Central nervous system involvement in Fabry disease is associated primarily with cerebrovascular dysfunction that contributes to a variety of neurological deficits ([6], reviewed in [5]). Prominent alterations in cerebral blood vessels, including stenosis of small vessels and enlargement of large vessels may occur either primary to glycosphingolipid accumulation or secondary to unresolved downstream signaling mechanisms and contribute to an increased risk and incidence for stroke in Fabry patients, in particular those that involve the vertebrobasilar system [6, 7]. White matter lesions are also common neuropathological findings, in addition to neuronal swelling, axonal degeneration and accumulation of ceroid lipofuscin [8–10].

The autophagy-lysosome pathway (ALP) is an important signaling pathway that maintains intracellular energy balance and in turn affects cell survival [11, 12]. Disruption of the ALP is a common hallmark of lysosomal storage diseases and several have documented alterations in the nervous system, which may contribute in part to the onset and progression of nervous system pathophysiology [13]. Disruption in the ALP has been documented previously in biopsies of Fabry disease patient muscle and kidney and *in vitro* in fibroblasts/lymphoblasts cultured from Fabry patients [14, 15]. However, whether the ALP is altered in Fabry disease brain has not been previously documented.

We have examined the CNS neuropathology resulting from α-Gal A deficiency by comparing brains from α-Gal A deficient vs. wild-type mice, using a well-established mouse model of Fabry disease with previous documented peripheral nervous system findings similar to those described in humans with Fabry disease [16–21]. We report widespread alterations of ALP-associated markers throughout the brains of α-Gal A-deficient mice. Such alterations are associated with vascular and parenchymal pathology as well as hindbrain axonal neurodegeneration, together suggesting that the ALP may play an important role in the development of CNS pathophysiology in Fabry disease.

## Methods

### Fabry disease mouse model

The α-Gal A gene-disrupted mouse, generated by insertion of a *neo* cassette in Exon 3 of the mouse *Gla* gene, lack α-Gal A enzymatic activity but otherwise live a normal lifespan [18]. Breeding pairs were obtained initially from the National Institutes of Health (Bethesda, MD) and in our colony were raised on a C57BL/6 background. Heterozygous (HET) females were bred with control males to maintain the mouse colony. Mutant male–female matings were performed to generate litters containing α-Gal A deficient mice for these studies. Mice were genotyped using the following primers: *Gla*-forward: 5′-ACTGGTATCCTGGCTCTATCC-3′; *Gla*-reverse: 5′-GATCTACGCCCCAGTCAGCAAATG-3′; Neo-reverse: 5′-TCCATCTGCACGAGACTAGT-3′, to indicate either a 550 bp product for control mice, or a 750 bp PCR product for α-Gal A deficient mice. Control C57BL/6 wild-type mice matched for age and strain were purchased from Charles River Laboratories, in association with the National Institute on Aging. Twenty- to 24-month-old C57BL/6 wild-type (+/o) and α-Gal A-deficient (-/0) male mice were used for this study. With the exception of electron microscopic analysis (*n* = 1 wild-type and α-Gal A-deficient mouse), results from all experiments were performed using male mice from at least three independent litters. “All animal experimentation conformed to UAB IACUC standards and Principles of laboratory animal care” (NIH publication No. 86–23, revised 1985) were followed.

### Specimen preparation

Mice were euthanized by exsanguination under isofluorane anesthesia, followed by trans-cardiac perfusion with PBS. Brains from perfused mice were removed, cut sagittally along the midline, and post-fixed in either 4% paraformaldehyde or Bouin’s fixative solution (71.5% saturated picric acid solution, 23.8% of a 37% w/v formaldehyde solution, 4.7% glacial acetic acid) for 48 hours at 4°C, followed by transfer to 70% EtOH. Hemi-brains were then processed and subsequently embedded in paraffin blocks and stored before sectioning.

Paraffin blocks were cooled on ice, cut on a Microm HM355S rotary microtome (Thermo Fisher Scientific, Waltham, MA) at a thickness of 6 μm, applied to Superfrost® Plus glass slides (12-550-15, Thermo Fisher Scientific, Waltham, MA), and baked overnight at 50°C. Before staining, the slides were deparaffinized in changes of CitriSolv® (22-143-975, Thermo Fisher Scientific, Waltham, MA) and 70% isopropanol. Antigen retrieval was accomplished by incubating in sodium citrate buffer (1.8% 0.1 M citric acid, 8.2% 0.1 M sodium citrate, in distilled water, pH 6.0) in a rice cooker for 30 minutes. The slides were blocked with PBS blocking buffer (1% BSA, 0.2% non-fat dry milk, and 0.3% Triton-X-100 in PBS) for 30 minutes, and treated with the appropriate primary antibodies diluted in blocking buffer overnight at 4°C. This was followed by incubation with secondary antibodies diluted in blocking buffer for 1 h at room temperature. The slides were then processed according to the following fluorescence or chromogenic IHC methods in preparation for imaging.

### Antibodies and reagents

Autophagosomes were labeled with a rabbit polyclonal antibody raised against mouse microtubule-associated protein light chain 3, or LC3 (Sigma, L7543, diluted 1:50,000). Lysosomes were labeled using rat-anti-mouse lysosome-associated membrane protein-1 (LAMP-1, University of Iowa Hybridoma Bank, clone 1D4B-s, diluted 1:2,000). Alpha-synuclein phosphorylated at serine-129 was labeled using rabbit-anti-mouse phosphorylated-α-synuclein (Abcam, ab168381, diluted 1:6,000). Ubiquitin was labeled using mouse-anti-bovine ubiquitin (Clone 6C1, Sigma, U 0508, diluted 1:10,000), a generous gift of Dr. Scott Wilson (UAB). Neuronal nuclei were labeled using mouse-anti-NeuN (Millipore, MAB377B, 1:5000). Secondary antibodies used were SuperPicture™ anti-rabbit polymeric antibody (Invitrogen, 87–9263, diluted 1:10) and ImmPress™ anti-mouse polymeric antibody (Vector Laboratories, MP-7402, diluted 1:50). Vascular endothelial cell surface labeling was done with fluorescein-tagged potato lectin (FPL, Vector Labs, FL-1161, diluted 1:1,000), obtained as a generous gift of Dr. Inga Kadisha (University of Alabama at Birmingham). Tyramide signal amplification (TSA) was used for detection. For fluorescence immunohistochemistry (IHC), TSA Plus-Cy3 (Perkin Elmer, NEL744E001KT) and TSA Plus-FITC (Perkin Elmer, NEL741001KT) were used. For chromogenic staining, biotin tyramide (Perkin Elmer, SAT700001EA) and avidin biotin complex reagent (ABC, Pierce, 32020), were used followed by development with 3,3′-diaminobenzidine tetrahydrochloride (DAB) substrate (Pierce, Rockford, IL), and nuclear counterstain with hematoxylin.

### Fluorescence IHC

Slides labeled for LC3, LAMP-1, and phosphorylated-α-synuclein antibodies intended for fluorescence were incubated in TSA Plus-Cy3 (diluted 1:1,500 in TSA amplification diluent) for 30 minutes at room temperature, and ubiquitin-labeled slides were incubated in TSA Plus-FITC (diluted 1:500 in TSA amplification diluent). For double labeling, slides were treated with hydrogen peroxide to block unused and endogenous peroxidases and blocked again before adding the second primary antibodies. All slides were counterstained with bis-benzimide (Sigma) nuclear stain (0.2ug/ml in PBS) for 10 minutes and mounted with Fluoromount G (SouthernBiotech, 0100–01) and 1.5 mm glass coverslips.

### Chromogenic IHC

Slides labeled with phosphorylated α-synuclein were incubated with biotin tyramide conjugate (diluted 1:400 in amplification diluent) for 10 minutes followed by ABC for 30 minutes. The slides were then developed using DAB peroxidase substrate for 10 minutes and quenched in water. After hematoxylin counterstain, the slides were mounted, coverslipped, and stored before imaging.

### Electron microscopy

Small mm^2^ sections of brain tissue were incubated overnight in “Half-Karnovsky’s fixative” (2% glutaraldehyde, 2.5% paraformaldehyde in 0.1 M cacodylate buffer with 2 mM Ca++ and 4 mM Mg++). Following fixation and including rinses between steps, the tissue was post-fixed with 1% osmium tetroxide in 0.1 M calcium carbonate buffer, dehydrated in an ethanol series up to 100% followed by 3 steps in propylene oxide. Finally the tissue was infiltrated and embedded over 2 days in EPON-812 epoxy resin. Sections were cut on a Reichert-Jung Ultracut-S ultra-microtome, stained with uranyl acetate and lead citrate.

### Imaging

Fluorescence imaging was performed on the Zeiss LSM 700 Confocal Microscope Platform (Carl Zeiss GmbH, Jena, Germany) or the Nikon A1 Confocal Microscope System (Nikon Instruments Inc., Melville, NY). Chromogenic imaging was performed on a Zeiss Axioskop microscope and captured using Zeiss Axiovision® software (Carl Zeiss GmbH, Jena, Germany). Ultrastructural images were obtained on the FEI Tecnai T12 Spirit transmission electron microscope at 80 kV (FEI, Hillsboro, OR) in the UAB High Resolution Imaging Facility. All images were subsequently processed in Adobe Photoshop® for presentation.

### Image and statistical analysis

Quantitative analysis of images was performed using ImageJ (National Institutes of Health, Bethesda, MD). Counting of phosphorylated α-synuclein aggregates and axonal spheroids was performed using the “cell counter” plugin. Phosphorylated-α-synuclein aggregates were considered if they exceeded 10 microns in diameter. Single label mean fluorescence intensity analysis for LC3 was performed using the “measure” command on background-subtracted images and recording the mean value. Co-localization analysis for phosphorylated α-synuclein with either LC3 or ubiquitin was performed using the “Coloc 2” plugin; the Costes p-value and thresholded Manders values were recorded as previously described [22]. Briefly, the Costes p-value is an indicator of the existence of co-localization within the field or region of interest. Threshold calculation accounts for background noise in each channel. The thresholded Manders analysis compares two color channels (probes) and provides a channel-specific measure of the number of pixels above threshold in one channel colocalizing with those in the other with a numerical range of 0 to 1, with 1 being complete co-localization. All co-localization analyses were calculated using data obtained from at least 2 separate fields from an n of 4 mice.

Statistical analysis for LC3 mean fluorescence intensity was performed using GraphPad Prism (GraphPad Software Inc., La Jolla, CA) and significance was determined at p < 0.05 using the Student’s t-test.

## Results

### Immunoreactivity of autophagy marker LC3 is increased throughout the brains of α-galactosidase A-deficient mice

**Figure 1:**
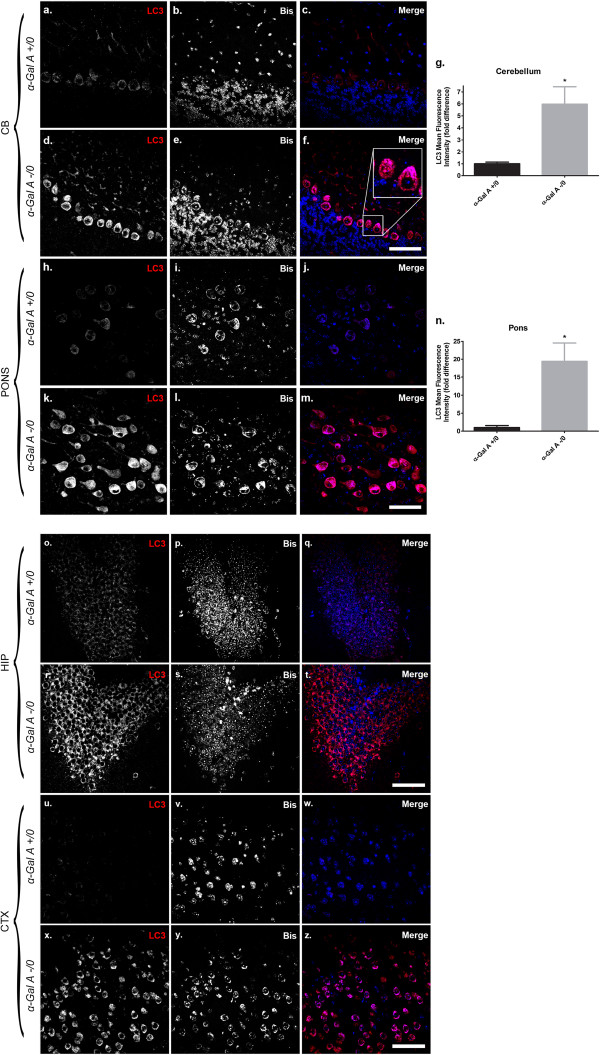
**LC3 levels are enhanced throughout α-Gal A-deficient mouse brain.** Sagittal brain sections from male 20- to 24-month-old α-Gal A +/0 **(a-c, h-j, o-q, u-w)** or -/0 **(d-f, k-m, r-t, x-z)** mice were immunolabeled with an antibody against LC3 **(a, d, h, k, o, r, u, x)** and counterstained with bis-benzimide **(b, e, i, l, p, s, v, y)**. Inset in panel **f** indicates LC3-positive puncta. The images shown were taken from the cerebellum (CB), pons (PONS), hippocampus (HIP), and cortex (CTX). Scale bars = 50 microns. Graphs in **(g and n)** show relative mean fluorescence intensity of LC3 signal normalized to averaged α-Gal A +/0 levels (n = 3) (*p < 0.05, Student’s t-test).

As α-Gal A deficiency is known to disrupt the ALP in skeletal and cardiac muscle and kidneys of patients with Fabry disease, we determined the extent to which the ALP is altered in the CNS by assessing brains of α-Gal A-deficient versus wild-type mice as a correlate to human CNS pathology [14, 15]. As a correlate to the age-dependent progression of Fabry disease, brain sections from aged (20-24-month-old) mice were probed with an antibody against microtubule-associated protein light-chain 3 (MAP-LC3 or LC3) to identify autophagosomes (Figure 1). In general, LC3 immunoreactivity was markedly higher throughout the gray matter of brains of α-Gal A-deficient mice (-/0) compared to those of age-matched wild-type control mice (+/0). Results in Figure 1 illustrate this relative increase in four distinct brain regions: the cerebellum (Figure 1a-f), pons (Figure 1h-m), hippocampus (Figure 1o-t) and cortex (Figure 1u-z). Quantification of the significant relative increase in mean fluorescence intensity of LC3 in the cerebellum and pons is shown in Figure 1g and Figure 1n, respectively.

Increases in cerebellar LC3 immunoreactivity were most striking in Purkinje cells from α-Gal A-deficient mice. The inset in Figure 1f highlights the punctate nature of LC3 staining, suggesting either the formation of LC3-positive autophagosomes or the accumulation of LC3-positive autophagic material. This inset is representative, as similar punctate staining patterns were noted throughout the areas surveyed. Enhanced LC3 immunoreactivity was also observed in the cerebellar molecular layer of α-Gal A-deficient (Figure 1d) versus wild-type (Figure 1a) mice. Increased LC3 immunoreactivity in α-Gal A-deficient mice was observed in dorsal and lateral aspects of the pons. Enhanced LC3 immunoreactivity in the hippocampus is illustrated in the dentate gyrus (Figure 1r), although increased immunoreactivity was observed in other sub-regions including the *Cornu Ammonis* (CA) regions (data not shown). Enhanced LC3 immunoreactivity was most apparent in layer 2 of the cortex from α-Gal A-deficient mice (Figure 1x), with minimal staining in layer 1 (data not shown). The area of the cortex depicted in Figure 1 (panels u-z) is from layer 2 of the somatomotor area.

### LAMP-1 immunoreactivity is markedly increased in both parenchymal and vascular regions of α-gal A-deficient mouse brain

**Figure 2:**
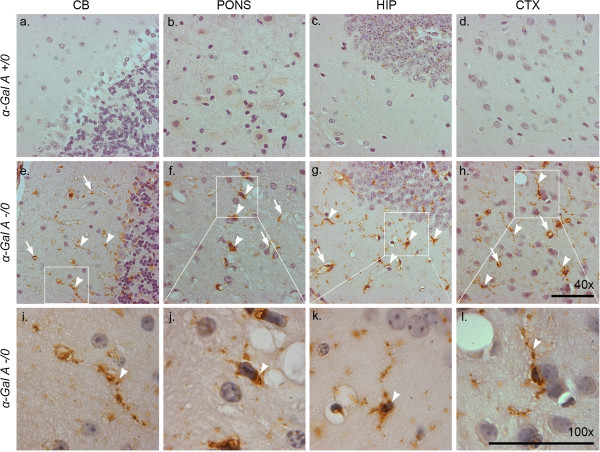
**Evidence of enhanced neuritic and perinuclear LAMP-1 levels.** Sagittal brain sections from male 20- to 24-month-old α-Gal A +/0 **(a-d)** or -/0 **(e-h)** mice were immunolabeled with an antibody against LAMP-1 and nuclei were counterstained with hematoxylin. Higher magnification areas of interest from α-Gal A -/0 sections are included for detail **(i-l)**. Arrowheads indicate areas of increased perinuclear **(f, j)** and neuritic **(e, g, h, I, k, l)** LAMP-1 immunoreactivity, while arrows indicate vascular LAMP-1 staining. The images shown were taken from the cerebellum, pons, hippocampus, and cortex as indicated. Scale bars = 50 microns.

**Figure 3:**
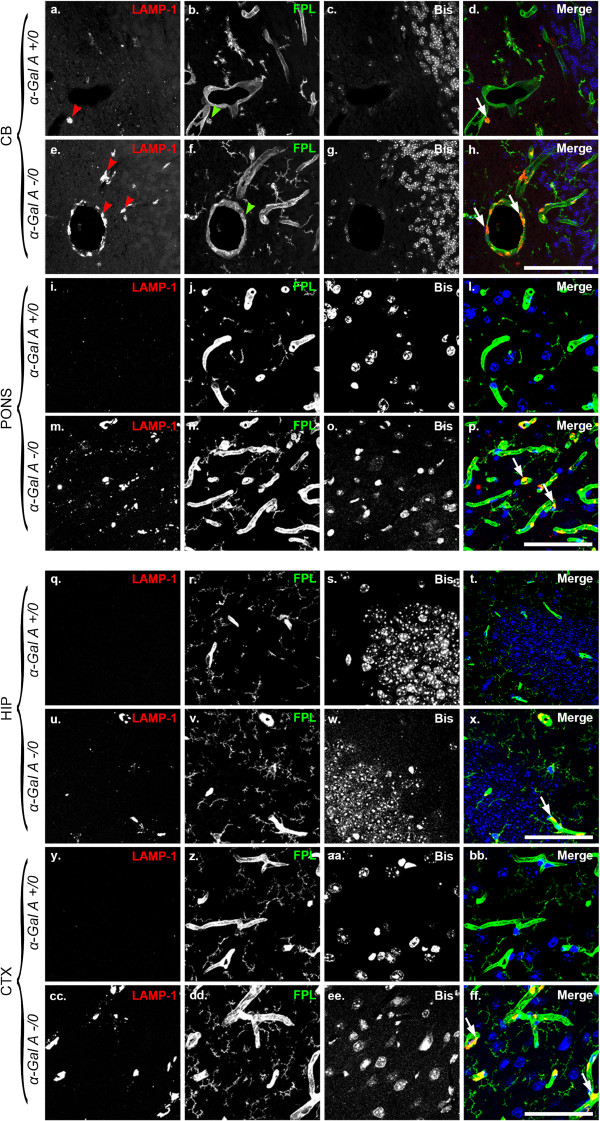
**LAMP-1 levels are enhanced throughout α-Gal A-deficient brain and exhibit vascular localization.** Sagittal brain sections from male 20- to 24-month-old α-Gal A +/0 **(a-d, i-l, q-t, y-bb)** or -/0 **(e-h, m-p, u-x, cc-ff)** mice were immunolabeled with an antibody against LAMP-1 **(a, e, i, m, q, u, y, cc)**, and fluorescent potato lectin **(b, f, j, n, r, v, z, dd)** and counterstained with bisbenzimide **(c, g, k, o, s, w, aa, ee)**. Red arrowheads **(a, e)** indicate areas of intense vascular LAMP-1 immuoreactivity and green arrowheads **(b, f)** indicate vascular staining in similar locations. White arrows **(d, h, p, x, ff)** indicate the preferential localization of LAMP-1-positive staining to the vascular endothelium. The images shown were taken from the cerebellum (CB), pons (PONS), hippocampus (HIP), and cortex (CTX) as indicated. Scale bars = 50 microns.

Increased levels of the lysosome marker LAMP-1 (lysosome-associated membrane protein-1) often accompany increases in LC3 in models of lysosomal storage diseases or models of induced lysosome dysfunction and suggest a compromise in autophagy completion [23–25]. To assess LAMP-1 immunoreactivity in α-Gal A-deficient mouse brain we first performed chromogenic detection (Figure 2). Consistent increases in LAMP-1 immunoreactivity were observed throughout the brains of α-Gal A-deficient versus wild-type mice, including but not limited to the cerebellum (Figure 2a, e, i), pons (Figure 2b, f, j), hippocampus (Figure 2c, g, k) and cortex (Figure 2d, h, l). Enhanced LAMP-1 immunoreactivity was localized to both perinuclear regions and neuritic processes in the parenchyma (arrowheads) in addition to an apparent vascular association with blood vessels (arrows). High magnification insets (Figure 2i-l) show in detail the perinuclear and neuritic staining patterns. To determine whether the increase in vascular LAMP-1 immunoreactivity in the brains of α-Gal A deficient mice was also localized to endothelial cells, brain sections were also double-labeled with an anti-LAMP-1 antibody and fluorescent potato lectin (FPL) (Figure 3), a fluorescein-tagged lectin that binds to cell-surface receptors on vascular endothelial cells [26]. There was no discernible difference detected in endothelial cell staining by FPL in α-Gal A deficient vs. wild-type mouse brain (indicated by green arrowheads in cerebellum, Figure 3b, f). However, enhanced LAMP-1 immunoreactivity in α-Gal A deficient mouse brains (indicated by red arrowheads in cerebellum, Figure 3a, e) co-localized remarkably with FPL labeling of endothelial cell surfaces (arrows, Figure 3h, p, x, ff).

### Ultrastructural analysis of α-galactosidase A-deficient mouse brain reveals accumulation of inclusions and lipopigment

**Figure 4:**
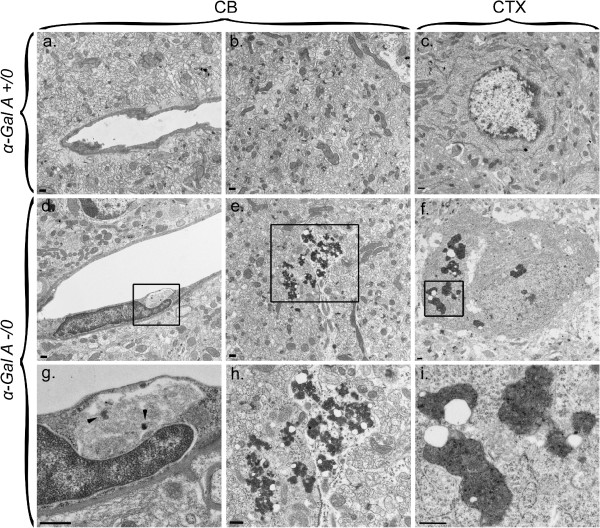
**Representative ultrastructural analysis of α-Gal A-deficient and wild-type mouse brain.** Ultra-thin sections of the cerebellum (CB; panels **a**, **b**, **d**, **e**, **g**, **h**) and cortex (CTX; panels **c**, **f**, **i**) of 21 month-old α-Gal A hemizygous (-/0; panels **d-i**) and α-Gal A wild-type (+/0; panels **a**-**c**) mice were prepared for electron microscopic analysis. Brain ultrastructure from the α-Gal A +/0 mouse exhibited normal appearing blood vessels **(panel a)** and perikarya **(panel b)** and neuronal cells with normal appearing nuclei and cytoplasm **(panel c)**. The endothelial cell lining a blood vessel in α-Gal A -/0 cerebellum contains a prominent inclusion (panel **d**; panel **g** represents inset box) exhibiting electron densities (arrowheads). In both cerebellum (panel **e**; panel **h** represents inset box) and cortex (panel **f**; panel **i** represents inset box) there appeared numerous lipopigment aggregates consisting of lipid droplets associated with electron-dense osmiophilic material. Scale bars = 500 nm.

As a correlate to our IHC results, we analyzed sections from the cerebellum and cortex of a 24 mo α-Gal A-deficient and wild-type mouse by electron microscopy to identify any ultrastructural abnormalities known to be associated with ALP deficiency [14]. Wild-type mice exhibited normal-appearing vasculature (Figure 4a) and parenchyma (Figure 4b) in the cerebellum and normal nuclei and cytoplasm in cortical neurons (Figure 4c). Representative images from α-Gal A-deficient mouse brain indicate a prominent electron-dense inclusion in the cytoplasm of a cerebellar endothelial cell that contains electron densities (Figure 4d, inset Figure 4g), and the presence of both cerebellar (Figure 4e, h) and cortical (Figure 4f, i) electron-dense osmiophilic inclusions consistent with accumulation of undigested lipopigment. We did not appreciate any accumulation of autophagosomes in α-Gal A-deficient mouse brain, suggesting that the increase in LC3 immunoreactivity observed by IHC in Figure 1 may be localized to lipid-containing aggregates of undigested autophagic substrate.

### Enhanced immunoreactivity for phosphorylated alpha-synuclein in α-gal A-deficient mouse brain is localized to areas of axonal degeneration

**Figure 5:**
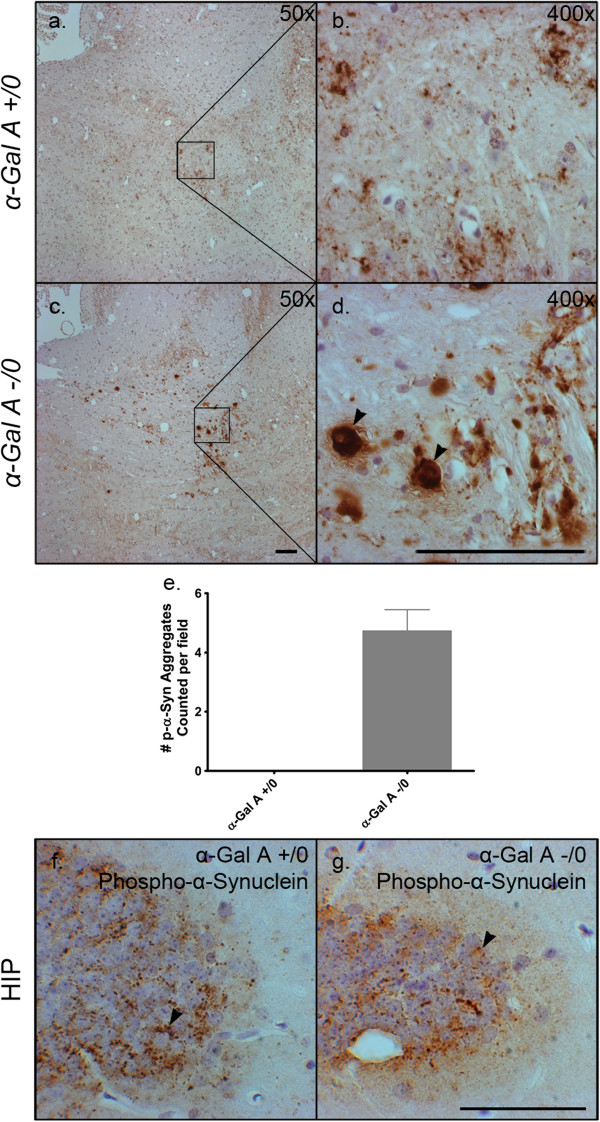
**Aggregates immunopositive for phosphorylated α-synuclein in the pons of α-Gal A-deficient brain.** Sagittal brain sections from male 20- to 24-month-old α-Gal A +/0 **(a, b, f)** or -/0 **(c, d, g)** mice were immunolabeled with an antibody against phosphorylated α-synuclein. Panels **a**-**d** show similar regions within the pons, and **(b and d)** are 8x magnifications of the boxes in **(a and c)**. Black arrowheads indicate areas of intense phosphorylated α-synuclein immunoreactivity **(d)**. Phosphorylated-α-synuclein aggregates above 10 microns in diameter were counted per field and graphed in panel **e**, which shows that none were present in α-Gal A +/0 pons while α-Gal A -/0 pons demonstrated an average of approximately 5 aggregates per field. Panels **(f and g)** show similar perinuclear phosphorylated α-synuclein staining patterns in the hippocampus between α-gal A +/0 **(f)** and -/0 **(g)** brains. Scale bars = 100 microns **(a-d)**, 50 microns **(f, g)**.

**Figure 6:**
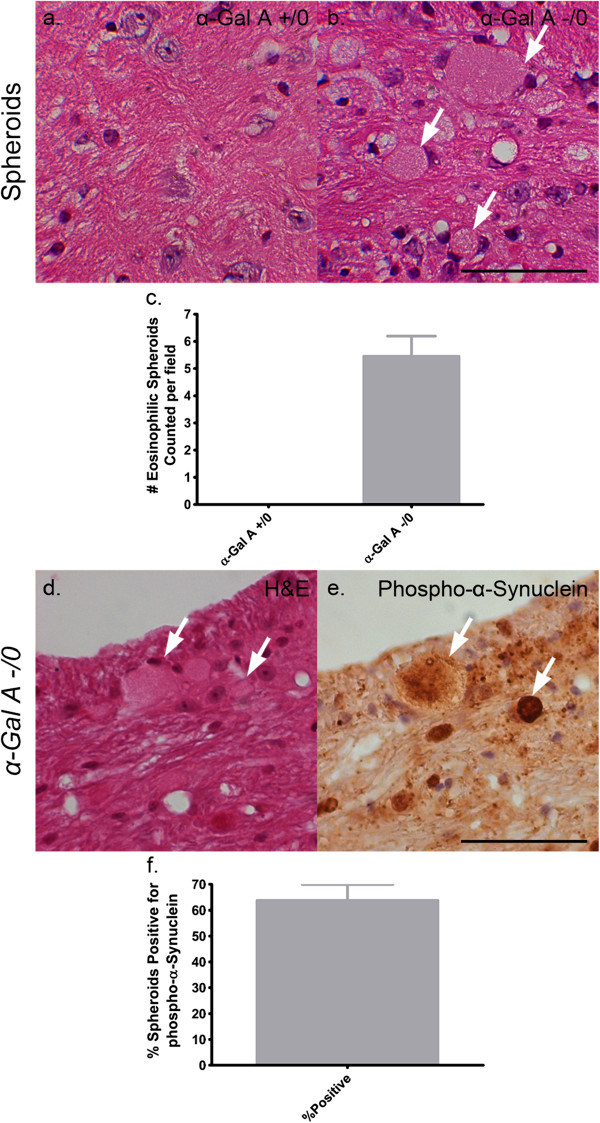
**Phosphorylated α-synuclein aggregates localized to axonal spheroids specific to the α-Gal A-deficient brain.** Sagittal brain sections from male 20- to 24-month-old α-Gal A +/0 **(a)** or -/0 **(b)** mice were stained with hematoxylin and eosin and serial sections from α-Gal A -/0 mice in the same group were stained with either hematoxylin and eosin **(d)** or an antibody against phosphorylated α-synuclein **(e)**. H&E staining indicates large eosinophilic axonal spheroids in α-gal A -/0 brains (white arrows, **b**) that are not evident in α-gal A +/0 **(a)**. This observation is quantified in **(c)**, comparing the number of axonal spheroids counted per field between α-Gal A +/0 and α-Gal A -/0 mice (n = 3). Panels d and e are serial sections demonstrating axonal spheroids (white arrows, **d**) that are immunopositive for phosphorylated-α-synuclein (white arrows, **e**) while panel **(f)** demonstrates the percentage of axonal spheroids that were positive for α-synuclein in serial sections (n = 3). Scale bars = 50 microns.

Alpha-synuclein is an ALP substrate and its aberrant accumulation has been previously documented following the pharmacological disruption of the ALP as well as in mouse models of lysosomal storage diseases with known ALP dysfunction [24, 27–31]. As the phosphorylated form of α-synuclein is closely associated with the neuropathogenesis of disease, we examined its distribution in the brains of α-Gal A-deficient mice (Figure 5) [32–34]. Immunohistochemical analysis for phosphorylated-α-synuclein revealed a noticeable accumulation of large interstitial aggregates of immunoreactivity localized to the pons white matter in α-Gal A-deficient mouse brain (Figure 5c, inset with arrowheads, Figure 5d). Immunoreactivity for phosphorylated-α-synuclein in the wild-type pons was less remarkable with the appearance of aggregates that were much smaller in size (Figure 5a, b). Quantification of aggregates in sections of a-Gal A deficient pons revealed an average of five per field that were greater than 10 microns in diameter (Figure 5e). Analysis of multiple sections from wild-type pons revealed a complete absence of these larger diameter aggregates. Perinuclear immunoreactivity of equal intensity was also observed for phosphorylated-α-synuclein in brains of wild-type and α-Gal A-deficient mice, as represented by images captured from the dentate gyrus sub-region of the hippocampus (Figure 5f, g). Further investigation of pontine neuropathology in α-Gal A-deficient mice by hematoxylin and eosin (H&E) analysis revealed the consistent appearance of large, swollen, eosinophilic axonal spheroids (Figure 6b, arrows) that indicate axonal degeneration and which are absent in wild-type mice (Figure 6a). Both observations are quantified in Figure 6c. In addition, histochemical analysis of the pons from serial sections of α-Gal A-deficient mouse brain indicated approximately 65 percent (Figure 6f) of areas of enhanced phosphorylated-α-synuclein immunoreactivity (arrows, Figure 6e) were within and adjacent to eosinophilic axonal spheroids (arrows, Figure 6d).

**Figure 7:**
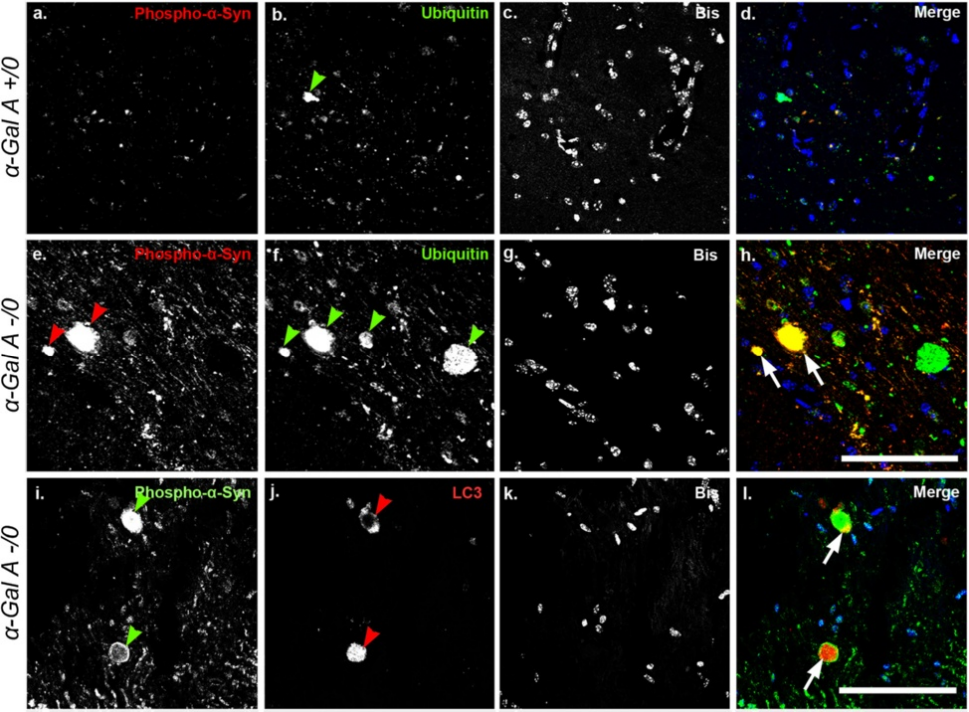
**Phosphorylated α-synuclein lesions in the α-Gal A-deficient pons co-localize with ubiquitin and LC3.** Sagittal brain sections from male 20- to 24-month-old α-Gal A +/0 **(a-d)** or -/0 **(b-l)** mice were immunolabeled with antibodies against phosphorylated α-synuclein **(a, e, i)** and ubiquitin **(b, f)** or LC3 **(j)**. Colored arrowheads indicate large lesions containing phosphorylated α-synuclein, ubiquitin, or LC3, and arrows indicate co-localization of large phosphorylated α-synuclein-containing lesions with either ubiquitin **(h)** or LC3 **(l)**. Co-localization analysis was performed using the thresholded Manders test. Manders co-localization between ubiquitin (M1) and phosphorylated-α-synuclein (M2) was calculated to be (tM1 = 0.506 and tM2 = 0.856, Costes p-value = 1, n = 3), indicating strong co-localization of phosphorylated-α-synuclein signal with ubiquitin in the pons of α-Gal A -/0 mice. Manders co-localization between phosphorylated-α-synuclein (M1) and LC3 (M2) was calculated to be (tM1 = 0.499 and tM2 = 0.765, Costes p-value = 0.958, n = 3), indicating strong co-localization of LC3 signal with phosphorylated-α-synuclein in the pons of α-Gal A -/0 mice. Scale bars = 50 microns.

As insoluble α-synuclein-containing aggregates are often associated with ubiquitin, we next performed double-label immunofluorescence analysis of phosphorylated-α-synuclein and ubiquitin (Figure 7a-h) to determine their relative degree of co-localization [34, 35]. In general, immunoreactivity for ubiquitin was noticeably greater in α-Gal A-deficient versus wild-type brain, as demonstrated in the pons by a relative enhancement of immunoreactive aggregate species (green arrowheads, Figure 7b, f), and paralleled increases in phosphorylated-α-synuclein observed in α-Gal A-deficient versus wild-type pons (red arrowheads, Figure 7a, e). Analysis of the merged images indicated that in α-Gal A-deficient pons, phosphorylated-α-synuclein aggregates were ubiquitin-positive (Figure 7h), whereas in the wild-type pons ubiquitin-positive aggregates were not immunoreactive for phosphorylated-α-synuclein (Figure 7d). Co-localization analysis of phosphorylated-α-synuclein and ubiquitin signals demonstrated co-localization to be both significant (Costes value of 1.0) and meaningful (tM1 = 0.506, tM2 = 0.856), such that 86% of pixels indicating phosphorylated-α-synuclein co-localized with those indicating ubiquitin.

It has been demonstrated previously that the disruption of the ALP contributes to axonal degeneration (reviewed in [36]). Thus double-label immunofluorescence was also performed to determine if phosphorylated-α-synuclein aggregates with spheroid-like morphology in the pons of α-Gal A-deficient mice co-localized with the autophagy marker LC3 (Figure 7i-l). LC3 immunoreactivity appeared in structures morphologically resembling axonal spheroids (red arrowheads, Figure 7j) and exhibited co-localization with phosphorylated-α-synuclein (Figure 7l), providing evidence that α-Gal A deficiency-induced axonal degeneration involves, in part, disruption of the ALP. Co-localization analysis of LC3 and phosphorylated-α-synuclein signals demonstrated co-localization to be both significant (Costes value of 0.958) and meaningful (tM1 = 0.499, tM2 = 0.765), where about 77% of pixels indicating LC3 co-localized with those containing phosphorylated-α-synuclein.

### Changes observed in α-gal A deficient mice are not associated with neuron loss

To address the possibility that autophagic and neuropathological changes observed in α-Gal A deficient mouse brain may be associated with changes with global changes in neuron density, we stained sections with an antibody against neuronal nuclei (NeuN) and counted positively-stained cells in each of 3 fields for each brain region (cortex, hippocampus, and pons) from α-Gal A +/0 (Additional file 1: Figure S1a-c) and α-Gal A -/0 (Additional file 1: Figure S1d-f) mouse brains (n = 3). Significant differences in numbers of NeuN positive neurons were not observed between α-Gal A +/0 and α-Gal A -/0 mouse brains (Additional file 1: Figure S1g).

## Discussion

Although α-Gal A deficiency has long been associated with nervous system dysfunction, a connection between central nervous system involvement and defects in the ALP has yet to be established in Fabry disease. We report widespread alterations of ALP-associated markers throughout the brains of α-Gal A-deficient mice. LC3, a marker of autophagic vacuoles, was substantially increased in multiple brain regions in connection with α-Gal A deficiency (Figure 1). LAMP-1, a marker of intact lysosomes was likewise increased throughout α-Gal A-deficient mouse brain, not only in neurons and neuritic processes, but also in vascular endothelial cells (Figures 2 and 3). Ultrastructural analysis revealed lipopigment-containing inclusions suggesting the accumulation of ALP byproduct (Figure 4). We also demonstrated the presence of large aggregate lesions of phosphorylated α-synuclein in the α-Gal A-deficient pons (Figure 5), which further co-localized with large axonal spheroids (Figure 6). Finally, we showed that α-synuclein-containing lesions in the pons also co-localized with ubiquitin and containing and LC3 (Figure 7). The present work thus illuminates a novel connection between distinct neuropathology and neurodegeneration (e.g. axonal spheroids) and alterations in the ALP, in a mouse model of α-Gal A deficiency.

Increased levels of ALP-associated markers clearly support a role for its disruption in the CNS of α-Gal A-deficient mice. Such increases would be predicted to indicate inhibition of macroautophagy completion secondary to lysosome dysfunction, a common feature of other lysosomal storage diseases [13, 23, 25, 37, 38]. In support of this argument, previous investigation of Fabry disease kidney, and cultured fibroblast/lymphoblasts from Fabry disease patients suggest that α-Gal A deficiency affects the ALP by inhibiting macroautophagy completion [14]. While our IHC results indicate increased punctate LC3 immunoreactivity (Figure 1), a relative lack of autophagosomes was observed by electron microscopic analysis (Figure 4). A possible explanation for this discrepancy may be the localization of LC3 with ceroid lipofuscin (Figure 4), considered to be an accumulation of autophagic material that is unable to be effectively degraded [37, 39]. However, recent *in vitro* analyses of α-Gal A deficiency indicated an increase in basal levels of LC3-II, the isoform of LC3 that is associated with double-membraned autophagosomes [23, 40], resulting possibly from aberrant autophagy induction [41]. Other studies have shown enhanced LC3 immunoreactivity in the absence of detectable autophagosomes, such as one describing VPS34 deficiency in cardiomyocytes [42], and another describing LC3-II localization to lipid droplets rather than autophagosomes [43]. As the availability of tissues for the present study was limited to specimens prepared for histochemical analyses, it will be useful in the future to assess levels of LC3-II by western blot analysis in frozen brain specimens obtained from α-Gal A-deficient versus wild-type mice, in addition to determining the relative state of autophagic flux to more accurately define the mechanisms by which α-Gal A regulates macroautophagy and in turn the degradation of autophagic substrate.

Although Fabry disease is considered first and foremost a vasculopathy by many investigators, our findings also demonstrate increases in parenchymal LC3 and LAMP-1 immunoreactivity localized to perinuclear and neuritic regions of neurons (Figures 1 and 2). Thus it is possible that ALP dysfunction in parenchymal tissues resulting from α-Gal A deficiency either occurs independently to, or results in part from endothelial cell dysfunction. Indeed, prominent findings of our study include enhanced endothelial cell immunoreactivity for LAMP-1 (Figure 3) and cytoplasmic endothelial cell inclusions (Figure 4). As the specimens used for this initial report were from relatively old mice aged 20–24 months of age, it will be interesting in future studies to examine the course of the onset and progression of vascular versus parenchymal pathology in the brains α-Gal A-deficient mice.

Our findings of aberrant phosphorylated-α-synuclein accumulation in α-Gal A-deficient mouse brain (Figures 5, 6 and 7) suggest that its metabolism relies on, at least in part, functional α-Gal A. Previous studies have indicated the importance of intact ALP function for the efficient degradation of α-synuclein, including several experimental models of lysosomal storage disorders [24, 28–31, 44–47]. Alpha-Gal A functions similarly to that of glucocerebrosidase, a soluble lysosomal enzyme that is mutated in Gaucher disease and is in the same sphingolipid catabolism pathway [48]. Glucocerebrosidase deficiency was shown recently to promote the accumulation of insoluble α-synuclein species [28]. In addition, mutations in the human *GBA* gene are also a strong genetic risk factor for Parkinson’s disease and glucocerebrosidase deficiency was reported recently in brain tissue from patients with Parkinson’s disease [28, 49, 50]. Although a link between Fabry disease and Parkinson’s disease has not been established, a case report of Parkinsonism in Fabry disease has been documented [51]. Alpha-Gal A deficiency has also been reported in leukocytes from patients with sporadic Parkinson’s disease, suggesting that α-Gal A dysfunction may regulate in part the pathogenesis of age-related neurodegenerative diseases like Parkinson’s, a possibility worthy of future investigation [52–54].

Accumulation of Gb_3_ has been documented previously in several tissues of α-Gal A-deficient mouse including the brain [19, 20]. Although ultrastructural analysis revealed prominent accumulation of lipoprotein in α-Gal A-deficient mouse brain, we were unable in the present study to accurately assess levels of glycosphingolipids due to the use of ethanol (which extracts lipids) to process brain tissues [55]. It has been reported previously that glucosylceramide, a glycosphingolipid that accumulates following glucocerebrosidase deficiency not only can promote the oligomerization of α-synuclein but can also induce neurotoxicity when added exogenously to neuronal cultures [28, 56]. These studies emphasize the importance for future investigations of α-Gal A-deficiency utilizing frozen brain tissue to accurately assess accumulation of glycosphingolipids and their relative contribution to the pathogenesis of Fabry-associated brain disease.

The detection of axonal spheroids in the pons of α-Gal A-deficient mouse brain (Figure 5) indicates axonal degeneration, a novel finding for α-Gal A-deficient mice and correlates with the previous observation in these mice of reduced axonal number and size in peripheral neurons [19]. CNS axonal spheroids were identified previously in other mouse models of lysosomal storage diseases and suggest impairments in axonal transport [27, 57, 58]. In addition, evidence of disrupted axonal transport was indicated by the co-localization of phosphorylated-α-synuclein immunoreactivity to axonal spheroids as shown previously in other models [27, 59]. Furthermore, the co-localization of phosphorylated-α-synuclein-containing aggregates/spheroids with ubiquitin and LC3 suggest the insoluble nature of α-synuclein and alterations in the ALP that may be causal for inducing axonal degeneration [36]. It is not known at this time why the pons of α-Gal A deficient mice was shown to be uniquely vulnerable to axonal degeneration. Previous investigations have indicated a particular sensitivity of the hindbrain in Fabry patients to vascular-associated ischemic attacks [5, 7, 60], thus the possibility exists for ischemic events to occur basally in α-Gal A-deficient mice that are localized to this brain region. Studies of peripheral nervous system function in α-Gal A deficient mice have identified reduced motor neuron conduction velocity, alterations in locomotor activity, and hypersensitivity to pain stimuli [19]. Whether alterations in the peripheral nervous system are connected to our observations in the central nervous system remains unclear. Future studies utilizing a rigorous stereological approach will help to elucidate neuronal pathways/tracts in the pons and other brain regions of α-Gal A-deficient mice that contribute to this neurodegenerative phenotype.

## Conclusion

In summary, our study reveals a consistent neuropathological and neurodegenerative phenotype existing in the brains of aged α-Gal A-deficient mice that is intimately associated with the disruption of the ALP. Future analysis of α-Gal A deficiency with respect to age, as well as rigorous biochemical and stereological analysis of brain specimens will all be useful in further delineating the onset and progression of CNS pathophysiology in these mice.

## Electronic supplementary material

Additional file 1: Figure S1: Neuron count was not significantly affected by α-Gal A deficiency. Sagittal brain sections from male 20- to 24-month-old α-Gal A +/0 (a-c) or -/0 (d-f) mice were immunolabeled with an antibody against neuronal nuclei (NeuN). NeuN Positive cells were counted from cortex, hippocampus, and pons, graphed (g), and statistical analysis was performed with GraphPad Prism (n = 3). (TIFF 6 MB)
