# Supplementary figures and images for: Autophagy-lysosome pathway associated neuropathology and axonal degeneration in the brains of alpha-galactosidase A-deficient mice
Source: Acta Neuropathol Commun. 2014 Feb 14;2:20. doi: 10.1186/2051-5960-2-20 (PMC3933238; doi:10.1186/2051-5960-2-20)

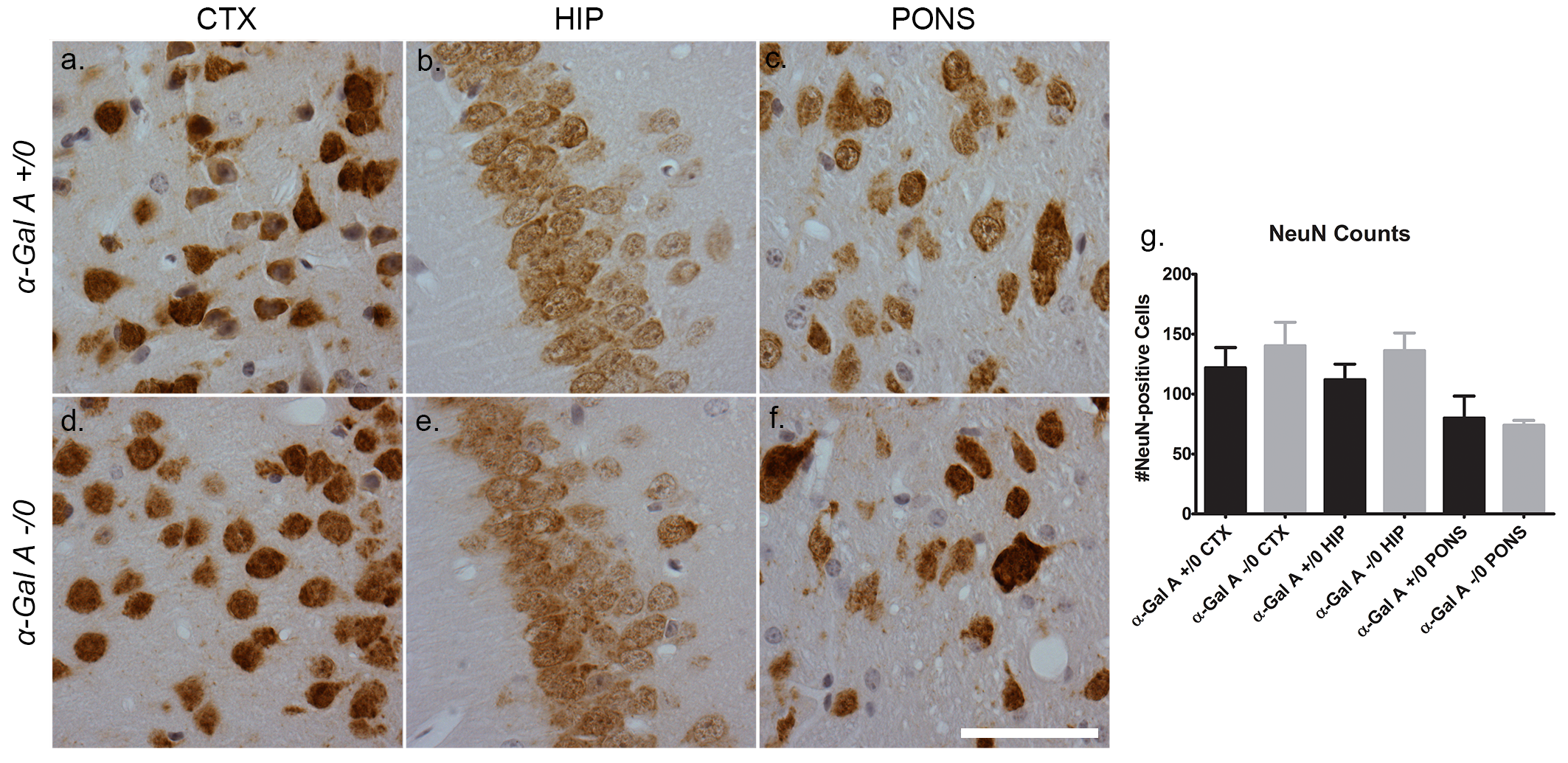

Supplement: Supplementary file 1 — Additional file 1: Figure S1: Neuron count was not significantly affected by α-Gal A deficiency. Sagittal brain sections from male 20- to 24-month-old α-Gal A +/0 (a-c) or -/0 (d-f) mice were immunolabeled with an antibody against neuronal nuclei (NeuN). NeuN Positive cells were counted from cortex, hippocampus, and pons, graphed (g), and statistical analysis was performed with GraphPad Prism (n = 3). (TIFF 6 MB) [file 40478_2013_96_MOESM1_ESM.tiff]
